# Effectiveness of a Web-Based Medication Education Course on Pregnant Women’s Medication Information Literacy and Decision Self-Efficacy: Randomized Controlled Trial

**DOI:** 10.2196/54148

**Published:** 2025-01-22

**Authors:** Suya Li, Hui-Jun Chen, Jie Zhou, Yi-Bei Zhouchen, Rong Wang, Jinyi Guo, Sharon R Redding, Yan-Qiong Ouyang

**Affiliations:** 1 School of Nursing Wuhan University Wuhan China; 2 Department of Obstetrics, Zhongnan Hospital of Wuhan University Wuhan China; 3 Renmin Hospital of Wuhan University Wuhan China; 4 Project HOPE Washington, DC United States

**Keywords:** decision self-efficacy, self-efficacy, decision efficacy, medication information literacy, information literacy, web-based medication education, medication education, web-based platforms, pregnant women, pregnancy, RCT, randomized controlled trial

## Abstract

**Background:**

Medication-related adverse events are common in pregnant women, and most are due to misunderstanding medication information. The identification of appropriate medication information sources requires adequate medical information literacy (MIL). It is important for pregnant women to comprehensively evaluate the risk of medication treatment, self-monitor their medication response, and actively participate in decision-making to reduce medication-related adverse events.

**Objective:**

This study aims to examine the effectiveness of a medication education course on a web-based platform in improving pregnant women’s MIL and decision self-efficacy.

**Methods:**

A randomized controlled trial was conducted. Pregnant women were recruited from January to June 2021 in the Department of Obstetrics and Gynecology of a large hospital in a major city in central China. A total of 108 participants were randomly divided into a control group (CG), which received routine prenatal care from nurses and physicians, and an intervention group (IG), which received an additional 3-week web-based medication education course based on the theory of planned behavior as part of routine prenatal care. Participants completed a Medication Information Literacy Scale and a decision self-efficacy questionnaire at baseline, upon completion of the intervention, and at a 4-week follow-up. Generalized estimation equations (GEE) were used to analyze the main effect (time and grouping) and interaction effect (grouping×time) of the 2 outcomes. The CONSORT-EHEALTH (V 1.6.1) checklist was used to guide the reporting of this randomized controlled trial.

**Results:**

A total of 91 pregnant women (48 in the IG and 43 in the CG) completed the questionnaires at the 3 time points. The results of GEE indicated that there was no statistically significant difference in time×group interactions of MIL between the 2 groups (*F*_2_=3.12; *P*=.21). The results of the main effect analysis showed that there were statistically significant differences in MIL between the 2 groups at T1 and T2 (*F*_1_=17.79; *P*<.001). Moreover, the results of GEE indicated that there was a significant difference in decision self-efficacy regarding the time factor, grouping factor, and time×group interactions (*F*_2_=21.98; *P*<.001). The results of the simple effect analysis indicated a statistically significant difference in decision self-efficacy between the 2 groups at T1 (*F*_1_=36.29; *P*<.001) and T2 (*F*_1_=36.27; *P*<.001) compared to T0. Results showed that MIL and decision self-efficacy in the IG were found to be significantly higher than those in the CG (*d*=0.81; *P*<.001 and *d*=1.26; *P*<.001, respectively), and they remained significantly improved at the 4-week follow-up (*d*=0.59; *P*<.001 and *d*=1.27; *P*<.001, respectively).

**Conclusions:**

Web-based medication education courses based on the theory of planned behavior can effectively improve pregnant women’s MIL and decision self-efficacy, and they can be used as supplementary education during routine prenatal care.

**Trial Registration:**

Chinese Clinical Trial Registry ChiCTR2100041817; https://www.chictr.org.cn/showproj.html?proj=66685

## Introduction

Pregnancy is a unique and sensitive period, accompanied by a series of temporary physiological and metabolic changes, which can directly affect the absorption, distribution, metabolism, and excretion of drugs [1]. With the prevalence of medication use during pregnancy and the occurrence of more and more safety accidents during this period, the safety of medications used during pregnancy has been paid increased attention [2,3]. Previous studies have shown that medication exposure during pregnancy is related to adverse pregnancy outcomes (eg, cumulative toxicities in pregnant women, birth defects, or preterm birth) and has long-term effects on fetal development [4].

Overestimating the teratogenic risk of medication [5,6], misinterpreting medication-related information [7], unconfirmed information and exaggeration of adverse medication reactions on the internet [8,9], and a large amount of terminology and vagueness of precautions in instructions [10] can lead to women’s confusion about medication use. This can increase the risk of optional medication withdrawal and exacerbate health conditions. Therefore, pregnant women not only need the ability to obtain appropriate medication information but also need to correctly interpret this information. They should be able to effectively distinguish the authenticity of medication information. This is influenced by medication information literacy (MIL) to some extent.

MIL refers to the information behavior related to medications, including the ability to seek, comprehend, identify, and use medication-related information [11]. This term combines medication literacy and information literacy, which is important for pregnant women to determine the risks of medication treatment and self-monitor medication reactions [12]. In addition, MIL represents the conceptualization of functional health literacy, emphasizing comprehensive, critical, proactive, and interactive capabilities and includes not only the demands identified, acquisition, understanding, evaluation, integration, and application of information but also medication information exchange, dosage calculation, and use of drug data to make smart health decisions [13]. Decision self-efficacy refers to self-confidence or a belief in one’s ability to make decisions, including shared decision-making [14]. It is an important factor affecting individual coping ability, individual choice, and the motivation and effort of trying to achieve a set of goals [15]. Pregnant women with low levels of MIL and decision self-efficacy have difficulties in correctly interpreting medication information and accurately implementing medication instructions, which may be a predictor of adverse medication events [16].

A previous survey showed that the MIL of pregnant women in China was generally low, but the demand for medication information was strong [11]. Therefore, it is urgent to carry out targeted interventions to improve the MIL of pregnant women.

**Figure 1:**
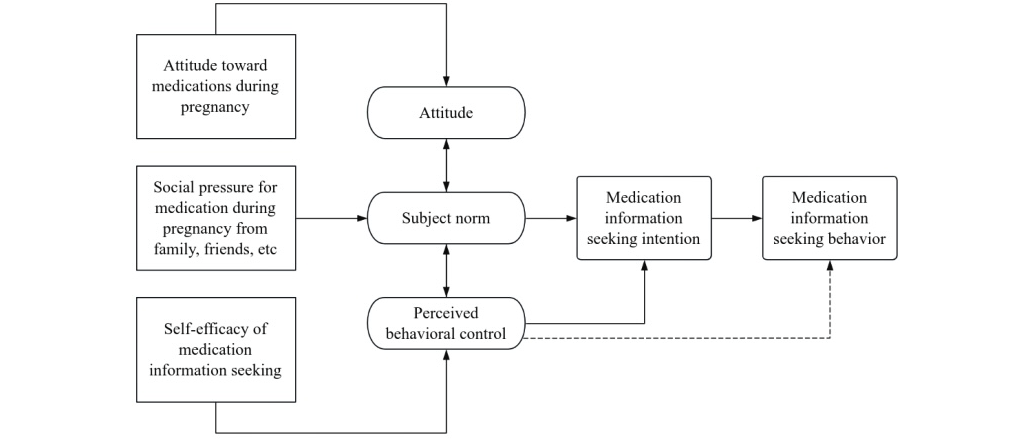
Application of the theory of planned behavior in this study.

The theory of planned behavior (TPB) is used to help predict and explain health behaviors and guide intervention programs [17] and is one of the most famous theories used in interpreting attitude-behavior relations [17,18]. According to TPB, behaviors are jointly determined by attitude toward the behavior, subjective norms, and perceived behavioral control. Previous studies have revealed the influencing factors of medication compliance and health literacy of pregnant women through the TPB [19-21]. The application of TPB in this study is shown in Figure 1.

During the COVID-19 pandemic, web-based education played an important role in meeting the needs of pregnant women [22]. To a certain extent, web-based education also provided protective isolation for pregnant women [23]. At present, WeChat (Tencent) has become a major social media web-based platform in China and has also received widespread attention from pregnant women [24]. Because of its convenience, timeliness, and intelligence, WeChat has made great efforts in the field of prenatal education related to blood glucose management [25], weight management [26], nutritional intervention [27], and breastfeeding promotion education [24]. Among pregnant women, the feasibility and effectiveness of health education based on the WeChat platform have been highlighted.

This study was undertaken to offer a health education course on the WeChat platform to empower pregnant women with the ability to inquire about, use, and evaluate medication information to actively participate in medical decisions and consciously resist adverse medication behaviors. Therefore, it is hypothesized that the web-based medication educational course would be effective in improving pregnant women’s MIL and decision self-efficacy.

## Methods

### Study Design

This study is a parallel randomized controlled trial delivering an additional 3-week web-based course about MIL to the intervention group (IG) with a 4-week follow-up. Pregnant women were recruited by convenience sampling from January to June 2021 in the Department of Obstetrics and Gynecology of a large hospital in a major city in central China. The study was registered in the Chinese Clinical Trial Registry (ChiCTR2100041817).

### Feasibility Trial

In order to explore the feasibility of a 3-week web-based course based on TPB, the researcher conveniently selected 10 pregnant women who met eligibility criteria in the department mentioned above from November to December 2020, for a feasibility trial. Based on the feedback of the pregnant women, the intervention program was further improved to the formal version. Participant’s completion of the interventions, attendance, and absences were recorded. Retention, compliance, and adherence rates were calculated.

The feasibility trial was well attended, all participants were engaged, interested in the course, and endorsed the intervention adaptations as culturally appropriate. All participants at session one indicated a desire to attend the remaining sessions, and 8 participants completed all the sessions, for an 80% adherence rate. However, only 7 of the women who attended all the sessions completed the questionnaire after the intervention, for a 70% retention rate. A number of barriers to attendance were identified as family obligations, the need to travel, and work obligations.

### Participants

#### Inclusion and Exclusion Criteria

Inclusion criteria were: primipara, at 24 weeks or less of gestation, reads and speaks Mandarin, is able to complete a questionnaire independently, agrees to take part in an education course, and has access to the WeChat platform. Exclusion criteria were: current participation in other research on similar topics and health care workers. In selecting the inclusion criterion of 24 weeks of gestation or less for primiparas, we considered multiple factors to ensure the validity and practicality of our study. One significant reason is that after 24 weeks, there is an increased likelihood of premature delivery, which could lead to participants dropping out of prenatal classes prematurely. This would not only disrupt the continuity of the intervention but also introduce bias by excluding those who might have experienced premature birth. By focusing on primiparas at or before 24 weeks, we aimed to minimize such dropouts and maintain a more homogeneous study population that could fully benefit from and complete the intervention program. This approach allowed us to accurately assess the intervention’s impact without any confounding effects of early termination due to premature.

#### Sample Size

The sample size was calculated according to the formula



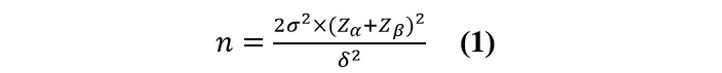



using a power of 0.8 and an α of 0.05, *Z_α_*=1.96 and *Z_β_*=0.84, as used in similar research [11], *σ*=8.22 and *δ*=6.00. The study required a minimum sample size of 29 (each group). Considering a 15% attrition rate, a minimum of 68 participants were needed for the study.

### Procedures

#### Recruitment

Women meeting eligibility criteria were invited to participate. The researcher explained the purpose of the study and informed that participation in the study was voluntary and that no prejudice would result from nonparticipation, termination, or withdrawal from the study. After all questions about the study were answered, participants were invited to sign an informed consent form.

#### Randomization and Blinding

Eligible participants were randomly assigned to the IG and the control group (CG) using a third-party organization [28]. The results of random allocation were placed in an opaque envelope. The researchers did not interfere with the random assignment, but, after allocation, the nature of the intervention made it difficult to blind participants and researchers.

#### Interventions

Participants in the CG received routine prenatal outpatient care from a physician. At the same time, they were contacted by the researchers upon initial enrollment in the study but received no further education courses.

Participants in the IG were offered an additional 3-week web-based course to improve MIL (Table S1 in Multimedia Appendix 1). The course was designed based on TPB [19] and used content from Drugs for Pregnant and Lactating Women [29] and the Risk Classification Systems of the US Food and Drug Administration [30]. The course focused on medication sensitivity during pregnancy, the popularity of essential medication, individual and social attitudes, and understanding, seeking, and evaluating medication information. The course arrangement was modified following the recommendations of pharmacists, obstetricians, and registered nurses. Feedback from study participants led to adjusting the course schedule to better meet the medication information needs of the women.

The web-based health education course consisted of 2 parts: an education module and a consultation module. The education module was delivered using the WeChat official account (Maternal and Child Guardian). Teaching strategies used in the course included both visual and auditory. Taking into account the concentration ability of pregnant women, each class was 15 minutes in length [31]. For the consultation module, the researchers established a WeChat group (individual account). Recent informational studies were shared with group participants every evening. A group chat was conducted weekly to review information and obtain feedback. In addition, participants were encouraged to contact researchers privately to ask questions, which would be answered within 24 hours. At follow-up, participants could review classes repeatedly. After 4 weeks of follow-up, an electronic questionnaire was sent to participants via the WeChat platform.

### Instrument

#### Sociodemographic Characteristics and Medication Use Questionnaire

Sociodemographic information included age, ethnicity, education level, location of residence, profession, household monthly income (in RMB), and gestational weeks. The medication use questionnaire included: (1) How many acute illnesses (such as fever and cold) did you have during pregnancy? (2) Have you ever used medications due to acute illnesses (such as fever and cold) during pregnancy? (3) How many chronic diseases did you experience during pregnancy (such as gestational hypertension, gestational diabetes, and coronary heart disease)? (4) Have you taken any medication for your chronic disease (such as gestational hypertension, gestational diabetes, and coronary heart disease) during pregnancy? (5) How many over-the-counter medications did you take during pregnancy?

#### Medication Information Literacy Scale

The Medication Information Literacy Scale (MILS) is designed to assess the MIL of pregnant women. It uses a 5-point Likert scale and consists of 22 items, including the 5 dimensions of medication information needs, medication information sources, medication information quality discrimination, medication information source awareness, and medication-taking behavior [11]. The MILS is scored by totaling the scores of all items. A higher score indicates a higher level of MIL. Pregnant women with scores below the threshold (80% of the total score) are considered to have insufficient MIL [32]. In this study, the Cronbach α coefficient of the total scale was 0.761 and the subdomains were approximately 0.564-0.824 [11].

#### Decision Self-Efficacy Scale

The decision self-efficacy scale, also called the decision confidence scale, is used to measure one’s confidence in making decisions, including the ability to take part in joint medical decisions. The scale consists of 11 items with a 5-point scoring system (0=not confident at all to 4=very confident) [14]. A higher score is associated with a higher level of self-efficacy [33]. The Cronbach α coefficient was 0.92 [33].

#### Data Collection and Analysis

Data collection was divided into 3 points: pretest at baseline (T0), immediately after the intervention (T1), and at 4 weeks follow-up (T2). The basic information questionnaire was only assessed at T0. The MILS and the decision self-efficacy scale were measured at T0, T1, and T2 using web-based questionnaires. To ensure the quality of the electronic questionnaires, the researchers stipulated that each participant had only one opportunity to submit the questionnaire, and incomplete questionnaires were not accepted.

Two-sample 2-tailed *t* test and chi-square test were used to compare the difference at baseline between the 2 groups. In addition, 1-way ANOVA analysis and independent 2-tailed *t* test were used to explore the influencing factors of MIL and decision self-efficacy. Data from the MIL and decision self-efficacy scales at T0, T1, and T2 were tested using generalized estimation equations (GEE). Data at T1 and T2 were added as the outcome and data at T0 were added as covariates. Time points and groups were added as independent variables. The effect of time, group, and time×group were analyzed. If the interaction effect of the time×group was not significant, the main effect analysis was performed; and if the interaction effect of the time×group was significant, the simple effect analysis was performed. Cohen *d* was used to evaluate the magnitude of the difference between the 2 groups. According to the standard established by Cohen, the large, medium, and small effects of *d* was divided into 0.8, 0.5, and 0.2 [34]. Missing values were replaced by the mean value if the missing items were less than 10% of the entire items in one scale, while the mean values were deleted if the missing items were more than 10% [35]. Data were entered in the SPSS (version 22.0; IBM Corp) and a 2-tailed *P* value of <.05 was considered statistically significant.

### Ethical Considerations

This study was approved by the Medical Research Committee of Wuhan University Medical College (2020YF0083), and informed consent was obtained before any study procedures. All participants were informed of the right to withdraw voluntarily. Data were maintained confidential and participants were allowed to complete the questionnaire or scale anonymously. Complying with the report’s comprehensiveness and authenticity, no data tampering or concealing of adverse results occurred. Maintaining the fairness principle, the CG was entitled to participate in the education course at the end of the study. Participants did not receive compensation for their participation.

## Results

### Baseline Characteristics of Participants

**Figure 2:**
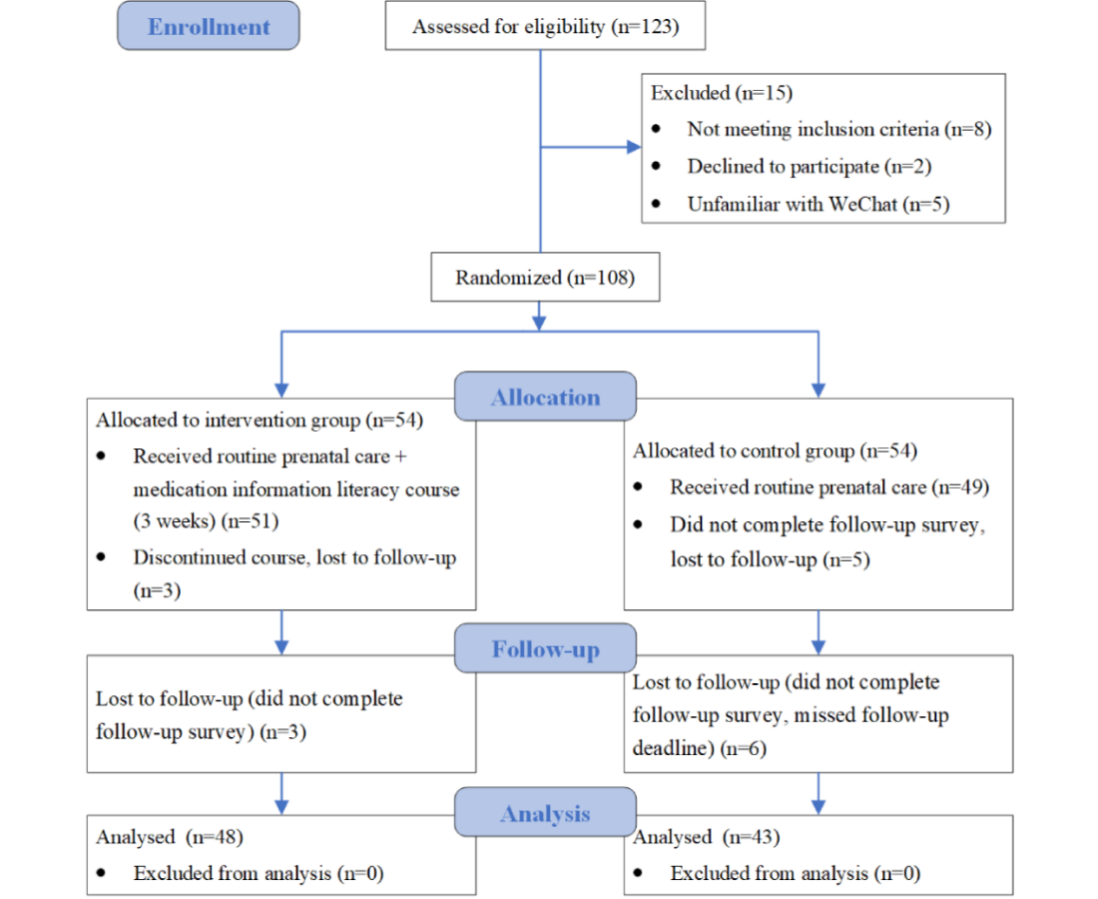
CONSORT diagram study flow.

In total, 108 participants completed the baseline test. Their average age was 29.46 (SD 5.03) years, and the average gestational week was 18.31 (SD 2.76). There was no significant difference between the 2 groups regarding sociodemographic characteristics, medication use, MIL, and decision self-efficacy at baseline (*P*>.05; Table S2 in Multimedia Appendix 1). Among the 2 groups, the average scores of MIL and decision self-efficacy were 81.00 (SD 10.94) and 22.31 (SD 4.43), respectively, and only 31% of participants had sufficient MIL. The implementation and data collection process are presented in Figure 2. The CONSORT-EHEALTH (V 1.6.1) checklist was used to guide the reporting of this randomized controlled trial (Multimedia Appendix 2).

Regarding the influencing factors, ANOVA analysis showed that there were statistically significant differences in the MIL and decision self-efficacy of participants related to education levels (*F*_107_=5.60; *P*=.001 and *F*_107_=3.03; *P*=.03, respectively) and gestational weeks (*F*_107_=0.80; *P*=.64 and *F*_107_=2.45; *P*=.01, respectively).

### Primary Outcome: MIL of Participants

At baseline, MIL scores showed no statistically significant difference between the IG and the CG (Table S3 in Multimedia Appendix 1). The results of GEE indicated that there was no statistically significant difference in time×group interactions of MIL between the 2 groups (*F*_2_=3.12; *P*=.21). Therefore, the main effects of the time and grouping factors were analyzed separately.

**Figure 3:**
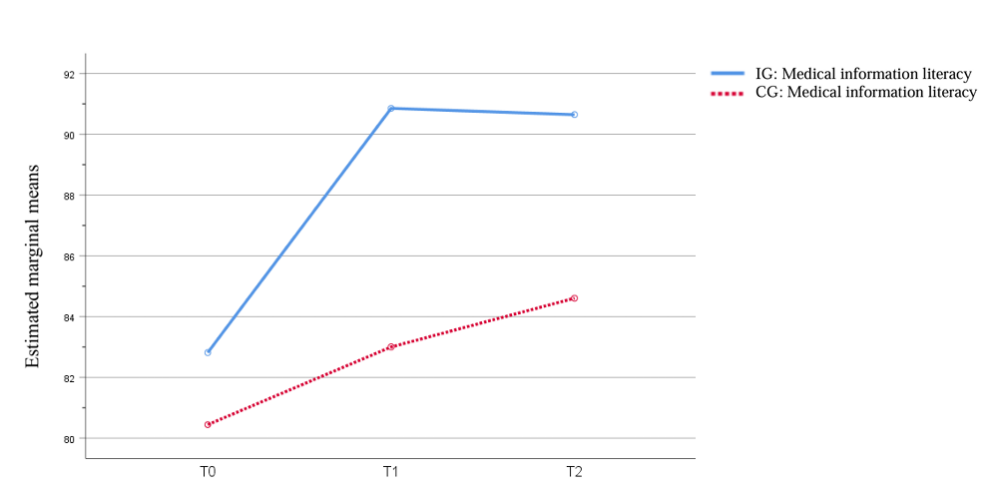
Estimated marginal means of medication information literacy. CG: control group; IG: intervention group.

The results of the main effect analysis showed that there were statistically significant differences in MIL between the 2 groups at T1 and T2 (*F*_1_=17.79; *P*<.001). The MIL of participants at T1 (mean difference 5.30, 95% CI 2.16-8.44; *P*<.001) and T2 (mean difference 6.00, 95% CI 2.80-9.20; *P*<.001) was significantly higher than at T0. Compared with the scores at T1, improvement in MIL in the follow-up phase was not as obvious, but it remained significantly improved compared to scores at T0 (Table S4 in Multimedia Appendix 1 and Figure 3).

### Secondary Outcome: Decision Self-Efficacy of Participants

**Figure 4:**
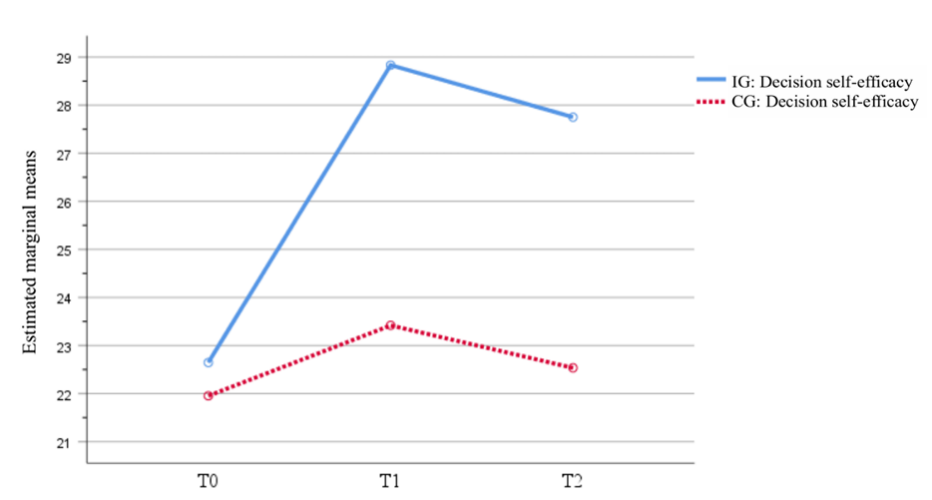
Estimated marginal means of decision self-efficacy. CG: control group; IG: intervention group.

At baseline, the scores on decision self-efficacy showed no statistically significant difference between the IG and the CG (Table S3 in Multimedia Appendix 1). The GEE results indicated that there was a significant difference in decision self-efficacy regarding the time factor, grouping factor, and time×group interactions (Table S4 in Multimedia Appendix 1 and Figure 4).

Since it was not meaningful to analyze the main effects of the time and grouping factors separately when the time×group interactions were significant (*F*_2_=21.98; *P*<.001), the simple effect of time and grouping were analyzed. Results displayed in Table S5 in Multimedia Appendix 1 indicated a statistically significant difference in decision self-efficacy between the 2 groups at T1 (*F*_1_=36.29; *P*<.001) and T2 (*F*_1_=36.27; *P*<.001) compared to T0; and there was a statistically significant difference between the self-efficacy of the IG at T1, T2, and T0 (*F*_1_=33.50; *P*<.001).

### The Effect Size of MIL and Decision Self-Efficacy

The results of Cohen *d* showed: (1) Compared with the CG, MIL of the IG had a large effect (*d*=0.81) at T1 and a medium effect (*d*=0.59) at T2. In addition, decision self-efficacy of the IG had a large effect (*d*=1.26 and *d*=1.27, respectively) at both T1 and T2. (2) Compared with T0, MIL had a moderate effect (*d*=0.76 and *d*=0.69, respectively) at T1 and T2, while decision self-efficacy showed a large effect (*d*=1.47 and *d*=1.24, respectively) at T1 and T2.

## Discussion

### Principal Findings

The results of this study suggest that the current web-based education based on the WeChat platform can improve the MIL and decision self-efficacy of pregnant women.

Studies estimating the effects of web-based medication education courses on MIL and decision self-efficacy in pregnant women are scarce. However, the current findings of the beneficial effects of web-based medication education in improving MIL and contributing to the safe use of medications are in line with a study that focused on older people [36]. In addition, it has been shown that decision self-efficacy can be improved through web-based education [37].

This study found that most pregnant women showed insufficient MIL which is in line with the study by Zhang et al [11], which revealed that the MIL of pregnant women was related to education level, residence, occupation, income, and gestational weeks. However, this research found that the MIL of pregnant women was related to their education level. This could be explained by the relatively small sample size. Current findings noted a significant improvement in MIL after 3 weeks of intervention.

Pregnant women’s negative beliefs about medications have been emphasized in previous studies [38-40]. Negative attitudes toward medications were also related to lower levels of pregnancy-related knowledge [41]. At the same time, excessive anxiety caused by overestimating the teratogenic risk of medication was one of the important reasons for low medication compliance and even self-discontinuation [5,38]. Furthermore, in the research of Barnes et al [42], the use of medication has been confirmed to be related to an individual’s religious and spiritual beliefs. According to previous findings, most respondents reported they needed more information about medication use and were concerned about inconsistent information from different sources [40,43]. Accordingly, this study developed a web-based medication education course in an attempt to change negative attitudes toward medication use among pregnant women, improve their medication beliefs, and strengthen their skills in obtaining, evaluating, and using medication information. The results of the study confirmed the effectiveness of this web-based course.

In this study, the average score of decision self-efficacy was much lower than that in Scaffidi et al’s [15] study, which might be interpreted that participants in this study were primiparous women. Compared with the decision self-efficacy of older people in previous studies [37,44], the decision self-efficacy of participants in this study was relatively low, assuming that the uniqueness and sensitivity of pregnancy make it more difficult for pregnant women to make medication-related decisions. Moreover, in this study, it was found that the decision self-efficacy of pregnant women was related to education level and gestational weeks, which is consistent with a previous study [15]. Lack of sufficient knowledge is the major factor affecting women’s decision-making [45,46]. The intervention in this study not only included the popularization of knowledge but also paid attention to the transformation of beliefs and improvement of skills to help pregnant women actively participate in medical decision-making, as well as express their concerns. After the 3-week course, the decision self-efficacy of the IG was significantly improved. The improvement of health literacy has a positive effect on perinatal women’s participation in medical decision-making [47]. The combination of effective communication skills and interactive health information technology is a potentially important exploration approach that can improve women’s enthusiasm for participating in medical decision-making [45]. The results of this study confirm the effectiveness of improving decision self-efficacy by increasing the MIL of pregnant women.

### Strengths

The overall attrition rate in this study was 16%. It may be related to the flexible class time and limiting each session to 15 minutes [31], which has been shown to improve the validity of research findings [48]. Overall, this study confirmed the effectiveness of the intervention content and the feasibility of web-based education, providing new ideas for improving MIL levels of pregnant women, especially those with limited access to health care resources in remote areas.

### Limitations

There are limitations to this study. Due to geographical restrictions, this study recruited pregnant women from only a tertiary hospital in a large city in central China and 71% of the participants were from cities, which might lead to sampling bias and further limit the external validity of the results. It could also limit the generalizability of findings to other areas. In addition, this study was carried out in the context of the COVID-19 pandemic, such that a potential impact on medication behavior cannot be excluded. Another limitation of this study centers on the researchers’ inability to adequately monitor the time that participants spent completing the learning modules. The researchers sent daily course updates to the women to encourage participation, help focus on content areas, and improve learning outcomes. Other teaching-learning strategies should be explored.

At the 4-week follow-up, MIL and decision self-efficacy were found to have declined slightly. Extending the web-based course over a longer period, as well as the follow-up evaluation, could improve the retention of content and test its effect on maternal and fetal outcomes. Another limitation may be that most of the participants did not take medications during the intervention, thus, there was no exploration of the relationship between medication adherence and MIL.

### Conclusions

Web-based medication education courses based on the TPB can improve pregnant women’s MIL and decision self-efficacy. After receiving web-based education, the average score of MIL of pregnant women rose significantly. As a function of health literacy, the improvement of MIL is expected to reduce the occurrence of adverse medication events in pregnant women. Future studies are needed to explore the long-term effects of similar courses, as well as content retention. A combination of web-based and offline education is also worthy of further exploration in the MIL and decision self-efficacy of pregnant women.

## Appendix

Multimedia Appendix 1 Supplementary tables.

Multimedia Appendix 2 CONSORT-eHEALTH checklist (V 1.6.1).

## Abbreviations

CG: control group
GEE: generalized estimation equations
IG: intervention group
MIL: medication information literacy
MILS: Medication Information Literacy Scale
TPB: theory of planned behavior

## Data Availability

The data sets generated during or analyzed during this study are not publicly available because the data also forms part of an ongoing study but are available from the corresponding author upon reasonable request.
