# Correction: Effectiveness of a Web-Based Medication Education Course on Pregnant Women’s Medication Information Literacy and Decision Self-Efficacy: Randomized Controlled Trial

**DOI:** 10.2196/91835

**Published:** 2026-01-26

**Authors:** Suya Li, Hui-Jun Chen, Jie Zhou, Yi-Bei Zhouchen, Rong Wang, Jinyi Guo, Sharon R Redding, Yan-Qiong Ouyang

**Affiliations:** 1 School of Nursing Wuhan University Wuhan China; 2 Department of Obstetrics, Zhongnan Hospital of Wuhan University Wuhan China; 3 Renmin Hospital of Wuhan University Wuhan China; 4 Project HOPE Washington, DC United States

In “Effectiveness of a Web-Based Medication Education Course on Pregnant Women’s Medication Information Literacy and Decision Self-Efficacy: Randomized Controlled Trial” [1], the authors noted an error in the affiliations.

The affiliation of authors SL, JZ, YBZ, JG, and YQO was previously shown as:

^1^Wuhan University

This affiliation has been revised to the following:

^1^School of Nursing, Wuhan University

The correction will appear in the online version of the paper on the JMIR Publications website, together with the publication of this correction notice. Because this was made after submission to PubMed, PubMed Central, and other full-text repositories, the corrected article has also been resubmitted to those repositories.
