# Supplementary material for: Effectiveness of a Web-Based Medication Education Course on Pregnant Women’s Medication Information Literacy and Decision Self-Efficacy: Randomized Controlled Trial
Source: J Med Internet Res. 2025 Jan 22;27:e54148. doi: 10.2196/54148 (PMC11799814; doi:10.2196/54148)
Supplement: Multimedia Appendix 1 [file jmir_v27i1e54148_app1.docx]

**Table S1. Curriculum of online education course guided by TPB**

| **TPB** | **Session** | **Contents** | Instructional format | Sequence |
| --- | --- | --- | --- | --- |
| Knowledge | Session 1  (Medication sensitivity during pregnancy) | Medication absorption | Text, pictures, audio and video | First week |
|  |  | Effects of medications at different stages of pregnancy | Text, pictures, audio and video |  |
|  |  | Can pregnant women receive COVID-19 vaccine? | Text, pictures, audio and video |  |
|  |  | Advantages and disadvantages of medication taken during pregnancy | Text and pictures |  |
|  |  | Review and discuss individual medication status during pregnancy | Group chat |  |
|  | Session 2  (Popularity of essential medication) | Common and contraindicated medication during pregnancy | Text and pictures |  |
|  |  | Identification of adverse medication reactions | Text, pictures, audio and video |  |
|  |  | Timing of medication usage | Text, pictures, audio and video |  |
|  |  | Review and discussion of prenatal supplements | Group chat |  |
| Attitude and subject norm | Session 3  (Individual and social attitudes) | Are all OTC (over the counter) medications safe? | Text and pictures | Second week |
|  |  | Common medication misconceptions and prejudices | Text and pictures |  |
|  |  | Appropriate use of prenatal supplements | Text, pictures, audio and video |  |
|  |  | Thalidomide incident | Case study, video |  |
|  |  | Review and discussion of personal views on medication during pregnancy | Group chat |  |
| Perceived behavioral control | Session 4  (Understanding medication information) | Common terminology found in medication labels | Text and pictures |  |
|  |  | Five-step method to interpret medication instructions | Text, pictures, audio and video |  |
|  |  | Common taboos about taking medication | Text, pictures, audio and video |  |
|  |  | Review and share information learned about reading medication labels or instructions | Group chat |  |
|  | Session 5  (Seeking medication information) | Sources of information | Text and pictures | Third week |
|  |  | Skills required | Text and pictures |  |
|  |  | Review and discuss experiences of finding medication information | Group chat |  |
|  | Session 6  (Evaluating medication information) | Evaluation of different sources | Text and pictures |  |
|  |  | Common types of fake medications and five ways to identify them | Text and pictures |  |
|  |  | Considerations in purchasing medication from online pharmacies | Text and pictures |  |
|  |  | Course review and summary | Text and pictures, group chat |  |
|  | Supplementary information | Medication during lactation | Text and pictures |  |

TPB: planned behavior theory

**Table S2. Socio-demographic characteristics and medication use** **of participants at baseline (n = 108)**

| Measure | Intervention group  (n=54)  mean (SD) / n (%) | Control group  (n=54)  mean (SD) / n (%) | *t* test (*df*) / Chi-square (*df*) | *P* value | |
| --- | --- | --- | --- | --- | --- |
| **Demographic information** |  |  |  |  | |
| Age (years) | 29.85 (4.87) | 29.07 (5.21) | .802^a^ | .42 | |
| Ethnicity |  |  | .911^b^ | .34 | |
| Han | 50 (93) | 47 (87) |  |  | |
| Minority | 4 (7) | 7 (13) |  |  | |
| Education |  |  | 2.578^b^ | .46 | |
| Junior high school or lower | 2 (4) | 2 (4) |  |  | |
| Senior high school | 21 (39) | 14 (26) |  |  | |
| Bachelor’s degree | 26 (48) | 34 (63) |  |  | |
| Master degree or higher | 5 (9) | 4 (7) |  |  | |
| Location of residence |  |  | .045^b^ | .83 | |
| Urban | 38 (70) | 39 (72) |  |  | |
| Rural | 16 (30) | 15 (28) |  |  | |
| Profession |  |  | 2.719^b^ | .74 | |
| Teacher | 3 (6) | 7 (13) |  |  | |
| Civil servant | 12 (22) | 14 (26) |  |  | |
| Corporate employee | 19 (35) | 16 (30) |  |  | |
| Self-employed | 8 (15) | 6 (11) |  |  | |
| Migrant worker | 10 (19) | 8 (15) |  |  | |
| Unemployed | 2 (4) | 3 (6) |  |  | |
| Household monthly income (in RMB) |  |  | .251^b^ | .97 | |
| <3000 | 4 (7) | 3 (6) |  |  | |
| 3001–5000 | 8 (15) | 9 (17) |  |  | |
| 5001–8000 | 18 (33) | 17 (31) |  |  | |
| >8000 | 24 (44) | 25 (46) |  |  | |
| Gestation (weeks) | 18.22 (2.77) | 18.39 (2.76) | -.313^a^ | .76 | |
|  |  |  |  |  | |
| **Medication use** |  |  |  |  | |
| How many acute illnesses (such as fever, cold, etc.) did you have during pregnancy? | | | 2.022^b^ | .36 |  |
| 0 | 26 (48) | 19 (35) |  |  |  |
| 1 | 22 (41) | 26 (48) |  |  |  |
| ≥2 | 6 (11) | 9 (17) |  |  |  |
| Have you ever used medication due to acute illnesses (such as fever, cold, etc.) during pregnancy? | | | .228^b^ | .63 |  |
| Yes | 10 (19) | 12 (22) |  |  |  |
| No | 44 (81) | 42 (78) |  |  |  |
| How many chronic diseases did you experience during pregnancy (such as gestational hypertension, gestational diabetes, coronary heart disease, etc.)? | | | .642^b^ | .73 |  |
| 0 | 40 (74) | 43 (80) |  |  |  |
| 1 | 12 (22) | 10 (19) |  |  |  |
| ≥2 | 2 (4) | 1 (2) |  |  |  |
| Have you taken any medication for your chronic disease (such as gestational hypertension, gestational diabetes, coronary heart disease, etc.) during pregnancy? | | | .121^b^ | .73 |  |
| Yes | 5 (9) | 4 (7) |  |  |  |
| No | 49 (91) | 50 (93) |  |  |  |
| How many over-the-counter (OTC) medications did you take during pregnancy? | | | 3.663^b^ | .16 |  |
| 0 | 50 (93) | 45 (83) |  |  |  |
| 1 | 4 (7) | 6 (11) |  |  |  |
| ≥2 | 0 (0) | 3 (6) |  |  |  |
|  |  |  |  |  |  |
| **Medication information literacy** | 82.30 (12.28) | 79.70(9.35) | 1.235^a^ | .22 |  |
| Sufficient MIL | 21 (39) | 13 (24) |  |  |  |
| Insufficient MIL | 33 (61) | 41 (76) |  |  |  |
|  |  |  |  |  |  |
| **Decision self-efficacy** | 22.65 (4.53) | 21.98 (4.35) | .781^a^ | .44 |  |

Note: ^a^ two-sample t-test, ^b^ Chi-square test, MIL = medication information literacy

**Table S3. Medication information literacy and decision self-efficacy of participants**

| Assessment points | IG (n=48)  Mean (SD) | CG (n=43)  Mean (SD) | *t* test (*df*) | *P* value |
| --- | --- | --- | --- | --- |
| MIL |  |  |  |  |
| T0 | 82.81 (12.07) | 80.44 (9.60) | 1.03 | .31 |
| T1 | 90.85 (8.97) | 83.00 (10.38) | 3.87 | <.001 |
| T2 | 90.65 (10.59) | 84.60 (10.06) | 2.79 | .007 |
| Decision self-efficacy |  |  |  |  |
| T0 | 22.65 (4.32) | 21.95 (4.36) | 0.77 | .44 |
| T1 | 28.83 (4.09) | 23.42 (4.49) | 6.02 | <.001 |
| T2 | 27.75 (3.93) | 22.53 (4.33) | 6.03 | <.001 |

Note: MIL = medication information literacy, IG = intervention group, CG = control group

**Table S4. Generalized Estimation Equations of outcomes**

| Source | Medication information literacy | | Decision self-efficacy | |
| --- | --- | --- | --- | --- |
|  | *F* test (*df*) | P value | *F* test (*df*) | *P value* |
| Time | 15.99 | <.001 | 47.29 | <.001 |
| Group | 17.79 | <.001 | 46.76 | <.001 |
| Time * group | 3.12 | .21 | 21.98 | <.001 |

**Table S5. Simple effect analysis of decision self-efficacy**

| Effect | Decision self-efficacy | |
| --- | --- | --- |
| Time (A)× Group (B) | *F* test (*df*) | *P value* |
| B by A1 | 0.58 | .45 |
| B by A2 | 36.29 | <.001 |
| B by A3 | 36.27 | <.001 |
| A by B1 | 33.50 | <.001 |
| A by B2 | 1.38 | .26 |

Note: A = time factor and B = grouping factor; A1 = pre-test at baseline (T0), A2 = post-test after the intervention (T1), A3 = post-test after four-week follow-up (T2), B1 = intervention group (IG) and B2 = control group (CG).
